# Characterization of pure mycelium materials from different mushroom-forming fungi

**DOI:** 10.1007/s10482-025-02133-5

**Published:** 2025-07-23

**Authors:** Jeroen G. van den Brandhof, Noortje Hansen, Chen Hou, Sander C. Broers, Martin Tegelaar, Han A. B. Wösten

**Affiliations:** Microbiology, Department of Biology, Utrecht University, Padualaan 8, 3584 CH Utrecht, The Netherlands

**Keywords:** Fungal material, Liquid shaken cultures, Mechanical properties, Mushroom forming fungi, Mycelium, Pure mycelium material

## Abstract

Pure mycelium materials (PMMs) are a sustainable alternative for a range of non-sustainable products such as textile, leather and meat. So far, properties of PMMs of different fungi have not been systematically assessed. Here, 11 mushroom-forming fungi, of which 10 isolated from nature, were grown in liquid shaken cultures. *Schizophyllum commune, Ganoderma resinaceum*, and *Trametes betulina* produced the highest biomass (8.62, 7.58, and 6.94 g L^−1^, respectively) when grown as mono-cultures in malt extract broth. Therefore, PMM properties were determined of mono-cultures, mixed-cultures, and co-cultures of these three fungi. The maximum tensile strength of the PMMs of the mono-cultures, mixed-cultures, and co-cultures of *S. commune*, *G. resinaceum*, and *T. betulina* did not show significant differences and ranged between 4.5 to 6.3 MPa. The elongation at break of the different PMMs was generally low and ranged between 0.8 and 1.6%. The Young’s modulus of the PMMs also showed relatively small differences ranging between 408 and 710 MPa. The *G. resinaceum* PMMs showed the lowest water uptake, while the *S. commune* mono-, mixed- and co-culture PMMs showed the highest water contact angle. Together, it is concluded that the properties of the mono-, mixed-, and co-cultures of *S. commune*, *G. resinaceum*, and *T. betulina* are not very different. These data suggest that the species of mushroom forming fungi does not have a major impact on PMM properties of biomass from liquid shaken cultures.

**Supplementary Information:**

The online version contains supplementary material available at 10.1007/s10482-025-02133-5.

## Introduction

Pure mycelium materials (PMMs) have a high potential as sustainable alternatives for a range of products including textile, leather, and meat. To this end, fungi are grown in liquid- or solid-state fermentation (Gandia et al. 2021). In the latter case, the PMMs are formed at the substrate-air interface as a mycelium skin (Jones et al. 2021; Pruckner et al. 2024) or are produced from mushrooms (van den Brandhof and Wösten 2022). Liquid-state fermentation is performed under both static and agitated conditions. Under static conditions, a sheet of mycelium forms at the water–air interface, known as liquid-state surface fermentation (Appels et al. 2018; Henning et al. 2022). By contrast, fungal biomass produced under agitated conditions is cast and dried, with or without prior homogenization of the mycelium (d’Errico et al. 2024; Wijayarathna et al. 2022).

Thus far, 28 mushroom-forming fungi have been used to make PMMs (van den Brandhof and Wösten 2022). The species most frequently used are *Ganoderma lucidum*, *Pleurotus ostreatus,* and *Schizophyllum commune* (van den Brandhof and Wösten 2022). PMMs of *G. lucidum* and *P. ostreatus* are characterised by a strength at break of 1.1 and 0.7 MPa and an elongation at break of 14 and 4%, respectively, when these fungi are grown on a cellulose film (Haneef et al. 2017). When grown on a combination of cellulose and potato-dextrose, these values are 0.8 and 1.1 MPa and 33 and 9% for *G. lucidum* and *P. ostreatus*, respectively (Haneef et al. 2017). PMM of *G. lucidum* from a solid-state fermentation has a tensile strength of 3.86 MPa and an elongation at break of around 4% (Pruckner et al. 2024), while these values are 0.3 MPa and 17.3%, respectively, for material from a liquid-state surface fermentation (LSSF) (Elsacker et al. 2023). PMM of *S. commune* from LSSF has a much higher tensile strength (6.5 and 9.5 MPa) but considerably lower elongation at break (1.4 and 1.3%) when grown in the dark and high CO_2_ or in the light and at high CO_2_, respectively (Appels et al. 2018). These examples show that the choice of fungus, fermentation method, and environmental growth conditions impact the properties of PMMs. Differences in PMM properties can even be obtained by deleting a single gene. A strain of *S. commune* in which the hydrophobin gene *sc3* has been deleted has an increased PMM density due to a highly reduced number of air voids. As a result, the tensile strength and the elongation at break of the Δ*sc3* strain is 40.4 MPa and 2.6% when the strain is grown in the light at high CO_2_ (Appels et al. 2018). Apart from using different species or strains or different growth conditions, PMM properties can be changed by physical and / or chemical processing during and / or after growth. For instance, compressing the mycelium can be used to change the PMM properties during growth (Pelletier et al. 2019), while crosslinkers and / or plasticizers (Appels et al. 2020; Wijayarathna et al. 2022; d’Errico et al. 2024) can be used after harvesting the fungal biomass. For instance, infiltration of *S. commune* PMM with 32% of the plasticizer glycerol lowers the tensile strength to 1.8 MPa and increases the elongation at break to 29.6% (Appels et al. 2020).

Notably, the properties of PMMs of different fungi have not been extensively studied. Here, 11 mushroom-forming fungi, of which 10 were isolated from nature, were examined for biomass production in liquid shaken cultures. PMM properties were determined of the three species with the highest biomass (*S. commune*, *Ganoderma resinaceum*, *Trametes betulina*) formed under these conditions. To this end, PMMs were produced from mono- and co-cultures as well as by mixing the mycelium of mono-cultures. Results indicate that the species does not have a major impact on PMM properties of biomass from liquid shaken cultures.

## Materials and methods

### Collection and identification of fungal species

Basidiocarp samples were collected in Utrecht and Zwolle (The Netherlands) and stored in plastic bags during transportation. The mushrooms were broken and 3–6 pieces of the inner part of the basidiocarp were placed in 55 mm diameter Petri dishes with sterilised wood chips. After incubation at 25 °C for 7–15 days, a colonised wood chip was transferred to malt extract agar (MEA) (CM0059, Oxoid Ltd, Basingstoke, United Kingdom) or potato dextrose agar (PDA) both supplemented with 50 μg ml^−1^ ampicillin. Mycelium was transferred to PDA without antibiotics and incubated at 25 °C until the plate was fully colonised. To extract genomic DNA, five needle plugs (⌀ 1 mm) from a colonised plate were resuspended in a 1.5 ml Eppendorf tube with 50 μl MilliQ water. Samples were exposed to 900 W for 1 min in a microwave. PCR was done using the lysate as template combining it with DreamTaq DNA Polymerase, DreamTaq buffer (ThermoFisher Scientific, MA, USA), and the ITS1 and ITS4 primers from the internal transcribed spacer (ITS) region or the LR0R and LR6 primers from the large subunit (LSU) region (Table S1). To identify specimen #30, PCR was also done on the lysate using primers EF1-1018F and EF1-1620R (Table S1) that anneal to the translation elongation factor 1α (TEF1) region. PCR consisted of 35 cycles (95 °C for 30 s, 55 °C for 30 s, and 72 °C for 60 s). PCR products were precipitated overnight at − 20 °C with isopropanol (1:1) and potassium acetate (1:10), and pelleted at 12.000 g for 10 min. The DNA was washed with 70% ethanol, dissolved in Milli-Q water, and sequenced at Macrogen Europe (Amsterdam, The Netherlands). Sequences were blasted against the MycoBank (Robert et al. 2005) and GenBank databases.

### Culturing

Strains isolated in this study and the monokaryotic *S. commune* strain 4.39 (*MAT*A41 *MAT*B41, CBS 341.81) were cultured for 7 − 10 days at 30 °C in the dark in 90 mm diameter Petri dishes containing 35 ml MEA. Five blocks (5 × 5 mm) of colonised MEA were transferred to a 50 ml Greiner tube with 20 ml malt extract broth (MEB) (CM0057, Oxoid Ltd, Basingstoke, United Kingdom) supplemented with 25 μg ml^−1^ chloramphenicol (hereafter, called MEB-Chl). Mycelium was grown for 6 days in the dark at 30 °C and 50 rpm. After adding 80 ml MEB-Chl, the cultures were homogenised for 30 s using a Waring Blender (Waring Laboratory, Torrington, England) and transferred to 250 ml Erlenmeyer flasks for a 24 h growth period in the dark at 30 °C and 200 rpm. These cultures were homogenised again for 30 s. Aliquots (100 mg wet weight mycelium ml^−1^) were used to inoculate cultures (see below).

### Quantification of biomass and rheology of the medium

Cultures were inoculated with 100 mg wet weight mycelium (50 mg of each species in co-cultures) and grown for 7 days at 30 °C in the dark at 200 rpm in 100 ml MEB-Chl in a 250 ml Erlenmeyer (*n* = 6 − 12). Mycelium was harvested by vacuum filtering using a pre-dried (1 h at 60 °C) and pre-weighed Melitta® coffee filter in a Büchner funnel (⌀ 120 mm) connected to a vacuum pump (Leybold, Divac 1.2L, Cologne, Germany). The biomass retained on the coffee filter was weighed after drying at 60 °C. Rheology of the spent medium was quantified using a MCR 300 rheometer with CP50 − 1 cone-plate (Anton Paar, Graz, Austria). Measurements were performed at 20 °C with 1 ml at a ramp log shear rate ($\dot{\gamma }$) ranging from 5 to 150 s^−1^ with 20 measuring points. Measurement data was evaluated using Rheoplus Software version 3.40. Initially, all data was analysed using the power law model (Eq. 1), where $\tau$ represents shear stress, $K$ the consistency index, $\dot{\gamma }$ shear rate and $n$ the flow behaviour index.

1 $$\tau =K\cdot {\dot{\gamma }}^{n}$$

For samples exhibiting shear-thinning behaviour (where $n$ < 1), the power law model was applied. For samples exhibiting Newtonian behaviour (where $n$ = 1 ± 0.03), the analysis was subsequently performed using the Newton equation (EQ. 2), where $\eta$ represents (shear) viscosity.

2 $$\tau =\eta \cdot \dot{\gamma }$$

### PMM sheet production

To produce PMM sheets, 2 L Erlenmeyers with 1200 ml MEB-Chl were inoculated with 200 mg wet weight macerated mycelium (100 mg of each species in the case of co-cultures). Cultures were grown for 7 days at 30 °C in the dark at 200 rpm. Pellet morphology of mono-cultures was assessed using a scanner (Epson Perfection V370 Photo) with 24-bit colour depth and a resolution of 1200 dpi, the scanner was covered with a black box to create a dark environment and provide a black background. Mycelium was filtered through nylon cheesecloth, washed with 2 − 3 volumes of demineralised water (dH_2_O), resuspended in 0.5 L dH_2_O and homogenised for 10 − 20 s with a blender (Triblade XL + Type HBM60, Kenwood Ltd, Havant, United Kingdom). To determine dry weight, 10 ml of the homogenate was dried at 60 °C. Based on the dry weight, a volume of blended biomass equivalent to 3 g dry weight (0.027 g dry weight cm^−2^) was used per sheet. To this end, the blended biomass was vacuum filtered using two layers of Miracloth (EMD Millipore Corp., Burlington, MA, USA) in a Büchner funnel (⌀ 120 mm) connected to a vacuum pump. Similarly, sheets were made by mixing mycelium (50:50) from mono-cultures. Mycelium sheets were covered with cellophane (Embalru, Nijverdal, Netherlands) and dried for 3 − 5 days at room temperature. Shrinkage after drying was measured by capturing images with a digital camera (Z6, Nikon, Tokyo, Japan). Surface area was analysed using ImageJ Fiji and compared to the Büchner funnel surface area.

### Mechanical properties of PMMs

PMM sheets were cut in dog bone shapes according to ISO standard 527 type 5A. Thickness was measured along the length of the sample at 3 random positions using a digital length gauge device (Heidenhain-Metro MT 1200, Traunreut, Germany). Samples were preconditioned at 25 °C and 50% relative humidity (RH) for at least 24 h. Tensile tests were performed at room temperature with a material testing machine (LS5, Lloyd Instruments/Ametek, PA, USA) using a 1 kN load cell and a preload force of 0.25 N with 2 mm min^−1^ test speed. The Young’s modulus was extracted with Rstudio using numerical differentiation.

### Scanning electron microscopy

Pieces of PMM (5 × 5 mm) or macerated mycelium were attached to an aluminium scanning electron microscopy (SEM) specimen stub with carbon adhesive disc (Agar Scientific, Essex, UK). Micrographs were acquired using the Phenom ProX Desktop SEM (Thermo Fisher Scientific, Waltham, USA) at 10 kV acceleration voltage. Fiber size was determined using 8-bit SEM images analysed with ImageJ Fiji and measured by manually determining the diameter of hyphae on 4 images per species (10 hyphae each) at the surface of the material.

### Water absorption and water contact angle of PMMs

Water absorption of PMMs was evaluated following ASTM D570. Samples (1 × 1 cm) were preconditioned at 25 °C and 50% RH for 24 h and submerged in dH_2_O. Weight increase was measured during 24 h after removing excess water with tissue paper. Samples were similarly preconditioned at 25 °C and 50% RH for water contact angle (WCA) measurements. Measurements were conducted at ambient temperature with an optical contact angle measuring and contour analysis system (OCA 15EC, DataPhysics Instruments, Filderstadt, Germany). A baseline was set manually before measuring the WCA of 5 μl MilliQ water. The WCA was measured 5–10 s after placing the droplet on the sample.

### Statistics

Differences in viscosity, consistency index and maximum tensile strength were assessed using ANOVA with Bonferroni post hoc corrections. In the case of heteroskedasticity a Welch’s ANOVA with Dunnett’s T3 post hoc was carried out to determine differences in biomass, pH, flow behaviour index, mass, shrinkage, thickness, density, elongation at break, Young’s modulus, water contact angle and water absorption after 24 h submersion. All analyses were carried out in IBM SPSS Statistics 29 using *p* ≤ 0.05.

## Results

### Biomass formation of mushroom forming fungi in liquid shaken cultures

10 mushroom-forming fungi were isolated from nature, identified by sequencing the ITS and LSU regions (Table 1), and grown in liquid shaken cultures. In general, mono-cultures of *S. commune* and *G.* *resinaceum* produced the highest dry weight biomass of 8.62 and 7.58 g L^−1^, respectively, when grown in MEB, followed by *T.* *betulina* with 6.94 g L^−1^ (Table 2). The lowest biomass was produced by *Fomes inzengae* with 2.43 g L^−1^. The biomass of co-culture combinations was typically below or between that of the two species grown individually (Table 2).

**Table 1:** Sequences of ITS and LSU regions for all isolated strains used in this study

ITS sequence	LSU sequence	Similarity
*Bjerkandera adusta* Specimen #42		
GCGGCGGCCTGGGTCTCATCCACTCTCAACTTCTGTGCACTTTTCATAGGCCGGCTTGTGGGTGCGTTCGCGCACTTGTAGGTGTCGGGCTTATGCTTTACTACAAACGATTCAGTTTTAGAATGTCATACTTTGCTATAACGCAATTTATATACAACTTTCAGCAACGGATCTCTTGGCTCTCGCATCGATGAAGAACGCAGCGAAATGCGATAAGTAATGTGAATTGCAGAATTCAGTGAATCATCGAATCTTTGAACGCACCTTGCGCTCCTTGGTATTCCGAGGAGCATGCCTGTTTGAGTCTCATGGAATTCTCAACCTTCGGCTTTATTGACGAAGGCTTGGACTTGGAGGTCGTGCCGGCTCTCGTAGTCGGCTCCTCTGAAATGCATTAGTGCGAACGTTACCAGCCGCTTCAGCGTGATAATTATCTGCGTTGCTGTGGAGGGTATTCTAGTGTTCGCGCTTCTAACCGTCTTCGGACAAATTTCTGAACTCTGAGCTCAAATCATGTAGGACTACCCGCTGAACTTAAGCATATCATAAGCGGAGGAATGGCCCCACAATTAAAACCGCTATT	TAGTAACTGCGAGTGAAGCGGGAAAAGCTCAAATTTAAAATCTGGCGGTCTTCGGCCGTCCGAGTTGTAATCTGGAGAAGCGTTTTCCGCGCTGGACCGTGTACAAGTCTCTTGGAACAGAGCGTCATAGAGGGTGAGAATCCCGTCTTTGACACGGACTACCAGTGCTATGTGATGCGCTCTCGATGAGTCGAGTTGTTTGGGAATGCAGCTCAAAATGGGTGGTAAATTCCATCTAAAGCTAAATATTGGCGAGAGACCGATAGCGAACAAGTACCGTGAGGGAAAGATGAAAAGCACTTTGGAAAGAGAGTTAAACAGTACGTGAAATTGCTGAAAGGGAAACGATTGAAGTCAGTCGCGTGTGCTAGAACTCAGCCTTGCTTTTGCTTGGTGCATTTTCTAGTGTACGGGCCAGCATCAGTTTTGGCCGCCGGAAAAAGGCCTTGGGAATGTGGCACCTTCGGGTGTGTTATAGCCCTTGGTTGTATACGGTGGCTGGGACTGAGGAACATAGCATGCCTTTACGGCGGGGCTTCGGCCACCTTCATGCTTTGGATGCTGGCGTAATGGCTTTAAACGACCCGTCTTGAAACACGGACCAAGGAGTCTAACATGCCTGCGAGTGTTTGGGTGGAAAACCCGAGCGCGTAATGAAAGTGAAAGTTGGGACTTCTGTCGTGGAAGGCACCGACGCCCGGACCAGACCTTCTGTGACGGATCCGCGGTAGAGCATGGATGTTGGGACCCGAAAGATGGTGAACTATGCCTGAATAAGGTGAAGCCAGAGGAAACTCTTGGTGGAAGCTCGTAGCGATTCTGACGTGCAAATCGATCGTCAAATTTGGGTATAGGGGCGAAAGACTAATCGAACCATCTAGTAGCTGGTTCCTGCCGAAGTTTCCCTCAGGATAGCAGAAACTCGTATCAGATTTATGTGGTAAAGCGAATGATAAAGGCCTTGGGGTTGAAACAACCTT	ITS: 99.81% with OK310731 LSU: 100% with CNRMA13.244
*Chondrostereum purpureum* Specimen #2		
CTGACTTTTCGGAGTATGTGCTCGCCTTTTGTAACTTACTACTTTTCCACTTGTGAACCTTTGTAGACTATGAATGAAATAAACTCATCTGAAAATGGTGGTTAAGAGAGGTTTGTTCTTACAGAGCATCCTTTTGTTAGTAGTTCATAGTCTATGTTATATTTACCCTTGATTGAATGTTAATGAATGTCATTTTACTGGTCTTTGACCTTTAAACTTAATACAACTTTCAGCAACGGATCTCTTGGCTCTCGCATCGATGAAGAACGCAGCGAAATGCGATAAGTAATGTGAATTGCAGAATTCAGTGAATCATCGAATCTTTGAACGCACCTTGCGCTCCTTGGTATTCCGAGGAGCATGCCTGTTTGAGTGTCATTAAATTCTCAACCTTATCAGTTTTTTATTAAATTGGTTCAAGGCTTGGATTTTGGGAGTTGCAGGCTTCTCTTTTGAAGTCAGCTCTTCTTAAATGTATTAGTGGAGACTTGTAACCGTCGCCTTGGTGTGATAATTATCTGCGCCTTGGTGTATTGGTGACTAGTAATGTCTTTGCTTATAACAGTCCATTAGATTGGACAATCACTTTATGACTTTTGACCTCAAATCAGGTAGGACTACCCGCTGAAC	TCCAGTCAAGAGACGGCTTGTTCTTACATATTTAAAGTTTGAGAATAGGTTAAGGTTGTTACAACCCCAAGGCCTCTAATCATTCGCTTTACCACATAAATCTGAAATGAGTTTCTGCTATCCTGAGGGAAACTTCGGCAGGAACCAGCTACTAGATGGTTCGATTAGTCTTTCGCCCCTATACCCAAATTCGACGATCGATTTGCACGTCAGAATCGCTACGAGCCTCCACCAGAGTTTCCTCTGGCTTCACCCTATTCAGGCATAGTTCACCATCTTTCGGGTCCCAACATACATGCTCTACCGCAGCTCCGTCACAAAAGGTCTGGGCTGGGCGTCGATGCACTCCATGACAGAGTTCTCAACTTTCACTTTCATTACGCACATGGGTTTTACACCCAAATACTCGCAGGCATGTTAGACTCCTTGGTCCGTGTTTCAAGACGGGTCAATTAAAGCCATTATGTCAACATCCTAAGCACGTACGTGGTTAAAAACCCGGCCTTGCGGCGTGCTGAATTCCTCAGTCCCAACTGTTATATACAATCAAGGGCTATAACACATCCGAAGATGCCACATTCCCCCAACCTTTATCTAACAGTCAAAACTGATGTTGACCCATCAACTAGGAAGTACACCAATCAAAAGAAAGGTTGATCCCCAGCTAATGCGACTGACTTCAAGCGTTTCCCTTTCAGCAATTTCACGTACTGTTTAACTCTCTTTCCAAAGTTCTTTT	ITS: 100% with CBS 350.53 LSU: 99.84% with CBS 352.53
*Fomes inzengae* Specimen #52		
TGCACGCCCTGCTCATCCACTCTACACCTGTGCACTTACTGTGGGATTTCAGGTGCGTCGCTTTAGCGGCGGCGTCACTCGGCCCACGTTTTCTTTACAAACTATTGAAGTAACAGAATGTTTATTGATGTAACGCATCTATAATACAACTTTCAGCAACGGATCTCTTGGCTCTCGCATCGATGAAGAACGCAGCGAAATGCGATAAGTAATGTGAATTGCAGAATTCAGTGAATCATCGAATCTTTGAACGCACCTTGCGCTCCTTGGTATTCCGAGGAGCATGCCTGTTTGAGTGTCATGAAATTCTCAACCTATAAACTTTTGCGGGTTTGTAGGGTTGGCTATTGGAGGCTTTTGCTGGCCCTCGTTTGAGTCAGCTCCTCTCAAATGCATTAGCTTGGTTCCTTGCGGATCGGCTGTCGGTGTGATAATGTCTACGCCGCGACCGTGAAGCGTTTGGAGAGCTTCTAATGGTCTCGTCAGAGACAGCTTTTATGAACTCTGACCTCAAATCAGGTAGGACTACCCGCTGAACTT	TAAGGTTGTTTCAACCCCAAGGCCTCTAATCATTCGCTTTACCACATAAATCTGATATGAGTTTCTGCTATCCTGAGGGAAACTTCGGCAGGAACCAGCTACTAGATGGTTCGATTAGTCTTTCGCCCCTATACCCAAATTTGACGATCGATTTGCACGTCAGAATCGCTACGAGCCTCCACCAGAGTTTCCTCTGGCTTCACCCTATTCAGGCATAGTTCACCATCTTTCGGGTCCCAACATACATGCTCTACCGCGGATCCGTCAGAGAACGTCAGGTCCGGGCGTCGATGCCCTCCACGACAGAGGTCTCAACTTTCACTTTCATTACGCGCTCGGGTTTTCCACCCAAACACTCGCAGGTAAGTTAGACTCCTTGGTCCGTGTTTCAAGACGGGTCGTTTAAAGCCATTATGCCAGCATCCTAAGCGCGAAAGTGGGCGAACCCCTGCCTTGCGGCGCGCTGCGTTCCTCGATCCCAACCGCCGCATGCGACTAGAGACTATAACACACCCGAAGGTGCCACATTTCCCTAGCCCTTTTCCGACGGTCAAAATCGATGCTGGCCCGTCGACCGGAAAATGCACCAAGCAAAAAGCAAGGCTGAGTCCCGGCCAACGCGACTGACTTCAAGCGTTTCCCTTTCAGCAATTTCACGTACTGTTTAACTCTCTTTCCAAAGTGCTTTTCATCTTTCCCTCACGGTACTTGTTCGCTATCGGTCTCTCGCCAATATTTAGCTTTAGATGGAATTTACCACCCATTTAGAGCTGCATTCCCAAACAACTCGACTCTTTGAGAGCGCATCACAAAGCACTGGGTAGTCCGTGTCAAAGACGGGATTCTCACCCTCTATGACGCTCTGTTCCA	ITS: 99.81% with OQ474914 (GenBank) LSU: 99.89% with OQ474914 (GenBank)
*Ganoderma australe* (syn. *G. adspersum*) Specimen #12		
TTGTAGCTGGCCTTACGAGGCATGTGCACGCCCTGCTCATCCGCTCCTACACCTGTGCACTTACTGTGGGTTTACGAGTCGCGAAACAGGCCCGTTCATTCGGGCTTGTGGAGCGCACTTGTTGCCTGCGTTTATCACAAACTCTATAAAGTATTAGAATGTGTATTGCGATGTAACGCATCTATATACAACTTTCAGCAACGGATCTCTTGGCTCTCGCATCGATGAAGAACGCAGCGAAATGCGATAAGTAATGTGAATTGCAGAATTCAGTGAATCATCGAATCTTTGAACGCACCTTGCGCTCCTTGGTATTCCGAGGAGCATGCCTGTTTGAGTGTCATGAAATCTTCAACTTACGAGCTTCTTGCGAGGTTTGTAGGGTTGGACTTGGAGGCTTGTCGGTCTTTAAAGGTCGGCTCCTCTTAAATGCATTAGCTTGGTTCCTTGCGGATCGGGTTGTCGGTGTGATAATGTCTACGCCGCGACCGTGAAGCGTTTGGCAAGCTTCTAACCGTCTCGGTATAGAGACAAGTTTTATGACCTCTGACCTCAAATCAGGTAGGACTACCCGCTGAAC	TCCGAGCGGTCCAATCAAGCGACGGCTCGTTCTTACATATTTAAAGTTTGAGAATAGGTTAAGGTTGTTTCAACCCCAAGGCCTCTAATCATTCGCTTTACCACATAAATCTGATAATGAGTTTCTGCTATCCTGAGGGAAACTTCGGCAGGAACCAGCTACTAGATGGTTCGATTAGTCTTTCGCCCCTATACCCAAATTTGACGATCGATTTGCACGTCAGAATCGCTACGAGCCTCCACCAGAGTTTCCTCTGGCTTCACCCTATTCAGGCATAGTTCACCATCTTTCGGGTCCCAACATACATGCTCTACCGCGGATCCGTCAGAGAACGTCTGGTCCGGGCGTCGATGCTCCCCACGACAGGGATCTCAACTTTCACTTTCATTACGCGCTCGGGTTTACCACCCAAACACTTGCAGGTATGTTAGACTCCTTGGTCCGTGTTTCAAGACGGGTCGTTTAAAGCCATTATGCCAGCATCCTAAGCGCGAAAGTGGGCGAACCCCTGCCTTGCGGCGCGCTGCGTTCCTCGATCCCAACCGCCGTATGCGACCAGAGTCTATAACACACCCCGAGGTGCCACATTAACTCCAGCCCTTTTCCGACGGTCAAAATCGATGCTGACCCGTCATCCGGAAAGTGCACCAAGCAAAAGCAAGGCTGAGTTCCGGACAACGCGACTGACTTCAAGCGTTTCCCTTTCAGCAATTTCACGTACTGTTTAACTCTCTATCCAA	ITS: 100% with AM906055 LSU: 99.08% with CBS 128580
*Ganoderma resinaceum* Specimen #18		
TAGCTGGCCTTCCGAGGCATGTGCACGCCCTGCTCATCCACTCTACACCTGTGCACTTACTGTGGGTTCCAGACGTTGTGAAGCGGGCTCTTTACGGGGCTTGTAAAGCGGCGTGCCTGTGCCTGCGTTTATCACAAACTCTATAAAGTATTAGAATGTGTATTGCGATGTAACGCATCTATATACAACTTTCAGCAACGGATCTCTTGGCTCTCGCATCGATGAAGAACGCAGCGAAATGCGATAAGTAATGTGAATTGCAGAATTCAGTGAATCATCGAATCTTTGAACGCACCTTGCGCTCCTTGGTATTCCGAGGAGCATGCCTGTTTGAGTGTCATGAAATCTTCAACTTACAGACCTTTGCGGGTTTGTAGGCTTGGACTTTGGAGGCTTGTCGGCCGTGTTTCGGTCGGCTCCTCTTAAATGTATTAGCTTGATTCCTTGCGGATCGGCTCTCGGTGTGATAATGTCTACGCCGTGACCCGTGAAGCGTTTTGGCGAGCTTCTAACCGTCTCGTTTGTGAGACAGCTTTATGACCTCTGACCTCAAATCAGGTAGGACTACCCGCTGAACTTA	AACTGCGAGTGAAGCGGGAAAAGCTCAAATTTAAAATCTGGCGGTCTTTGGCCGTCCGAGTTGTAGTCTGGAGAAGTGCTTTCCGCGCTGGACCGTGTATAAGTCTCTTGGAACAGAGCGTCATAGAGGGTGAGAATCCCGTCTTTGACACGGACTACCAGTGCTTTGTGATGCGCTCTCAAAGAGTCGAGTTGTTTGGGAATGCAGCTCAAAATGGGTGGTGAATTCCATCTAAAGCTAAATATTGGCGAGAGACCGATAGCGAACAAGTACCGTGAGGGAAAGATGAAAAGCACTTTGGAAAGAGAGTTAAACAGTACGTGAAATTGCTGAAAGGGAAACGCTTGAAGTCAGTCGCGTTGTCCGGAACTCAGCCTTGCTTTCGCTTGGTGCACTTTCCGGATGACGGGTCAGCATCGATTTTGACCGTCGGAAAAGGGCTGGAGTAATGTGGCACCTCCGGGTGTGTTATAGACTCTGGTCGCATACGGCGGTTGGGATCGAGGAACGCAGCGCGCCGCAAGGCAGGGGTTCGCCCACTTTCGCGCTTAGGATGCTGGCATAATGGCTTTAAACGACCCGTCTTGAAACACGGACCAAGGAGTCTAACATACCTGCGAGTGTTTGGGGTGGAGAACCCGAGCGCGTAATGAAAGTGAAAGTTGAGATCCCTGTCGTGGGGAGCATCGACGCCCGGACCTGACGTTCTCTGACGGATCCGCGGTAGAGCATGTATGTTGTGACCCGAAAGATGGTGAACTATGCCTGAATAGGGTGAAGCCAGAGGAAACTCTGGTGGAGGCTCGTATCGATTCTGACGTGCACATCGATCGTCAAATTTGAGTATAGGGGCGAAAGACTAATCGAACCATCTAGTAACTGGTTCCTGCCCAAGTTTCCCTCATGATAGCAGAGACTC	ITS: 100% with MT397406 LSU: 99.21% with CBS 220.36
*Pleurotus ostreatus* Specimen #19		
ATGTGCACGCTTCACTAGTCTTTCAACCACCTGTGAACTTTTGATAGATCTGTGAAGTCGTCTTTCAAGTCGTCAGACTTGGTTTGCTGGGATTTAAACGTCTCGGTGTGACAACACAGTCTATTTACTTAACACACCCCAAATGTATGTCTACGAATGTCATTTAATGGGCCTTGTGCCTATAAACCATAATACAACTTTCAACAACGGATCTCTTGGCTCTCGCATCGATGAAGAACGCAGCGAAATGCGATAAGTAATGTGAATTGCAGAATTCAGTGAATCATCGAATCTTTGAACGCACCTTGCGCCCCTTGGTATTCCGAGGGGCATGCCTGTTTGAGTGTCATTAAATTCTCAAACTCACTTTGGTTTTTTTC	AGTTTGAGAATAGGTTAAGGTTGTTTCAACCCCAAGGCCTCTAATCATTCGCTTTACCACATAAATCTGATATGAGTTTCTGCTATCCTGAGGGAAACTTCGGCAGGAACCAGCTACTAGATGGTTCGATTAGTCTTTCGCCCCTATACCCAAATTCGACGATCGATTTGCACGTCAGAATCGCTACGAGCCTCCACCAGAGTTTCCTCTGGCTTCGCCCTATTCAGGCATAGTTCACCATCTTTCGGGTCCCAACATACATGCTCCACCGCATATCCGTCCGTAAACTTCTGGTATGGGCGTCGGTGCTCCCCATGACAGGGATCCCAACTTTCACTTTCATTACGCGCTCGGGTTTTCCACCCAAACACTCGCAGGCATGTTAGACTCCTTGGTCCGTGTTTCAAGACGGGTCGATTAAAGCCATTTTGCCAGCATCCTAAGACAGCCTTGCGGCGCTGAGTTCCTCAGTCCCAGCCACTGTATCTGATCAAAGACTATAACACACCCGAAGGTGCCACATTTCTCTGACCTTTATCCAGCGACCAAAACTGATGCTGACCCATCAACCAGGAAGTACGCCTCACAAAAGTAAGGTTGATCCCTGGCAGACGCGACTGACTTCAAGCGTTTCCCTTTCAACAATTTCACGTACTGTTTAACTCTCTTTCCAAAGTTCTTTTCATCTTTCCCTCACGGTACTTGTTCGCTATCGGTCTC	ITS: 100% with CBS 342.69 LSU: 100% with CBS 373.51
*Phlebia tremellosa* Specimen #21		
CGCTGGTCCCGTTCTTAACAGAGTCGGGACATGTGCACACCTGGCTTCATTCCACTCTTCAACCCCTGTGCACTTGTTGTAGGTTTCAGGTCGGACGCGGGGCTTTCGACTTCATCGTCGAGAGCCTCAAGGAAAGCCTGAGCCTATGTTTTACCATACACACGCTTCAGTTATAGAATGTAAACATCGCGTATAACGCATTTTAAATACAACTTTCAGCAACGGATCTCTTGGCTCTCGCATCGATGAAGAACGCAGCGAAATGCGATAAGTAATGTGAATTGCAGAATTCAGTGAATCATCGAATCTTTGAACGCACCTTGCGCTCCTTGGTATTCCGAGGAGCATGCCTGTTTGAGTGTCATGGAATTCTCAACCTTTGAAAGCTTTTATCGGTTCTCAAAGGCTTGGACTTGGAGGTCGTGTCGGCTCTGCAAAGAATATCGACTCCTCTGAAACGTATCAGTGCGAACCTTTACGGATCGTCTTCGGTGTGATAAACGTCTACGCCGTGGTCGTGAAGTATGATCGTGTCCGTGCTTCTAACCGTCTCCTTTGCGAGACAAAAACTGTGTTACTTACTATAAGTAACATAGGCATTGACAATCTGACCTCAAATCAGGTAGGACT	ATTAAGTGACGGCTCGTTCTTACATATTTAAAGTTTGAGAATAGGTTAAGGTTGTTTCAACCCCAAGGCCTCTAATCATTCGCTTTACCACATAAATCTGATACGAGTTTCTGCTATCCTGAGGGAAACTTCGGCAGGAACCAGCTACTAGATGGTTCGATTAGTCTTTCGCCCCTATACCCAAATTTGACGATCGATTTGCACGTCAGAATCGCTACGAGCCTCCACCAGAGTTTCCTCTGGCTTCACCCTATTCAGGCATAGTTCACCATCTTTCGGGTCCCAACATACATGCTCTACCGCATATCCGTCACAGAAGGTCTGGTATGGGCGTCGGTGCTCCCCACGACAGGGATCCCAACTTTCACTTTCATTACGCGCTCGGGTTTTCCACCCAAACACTCGCATGCATGTTAGACTCCTTGGTCCGTGTTTCAAGACGGGTCGTTTAAAGCCATTACGCCAGCATCCTAAGCACGTACGTGGGCGAACCCCGGCCAAAAGGCGTGCTGCGGTCCTCAGTCCCGACCGTCGTATACGACCGACGGCTATAACACACCCGGAGGTGCCACATTCCGAAGACCTTTTTCCGACAGTCAAAACTGATGCTGGCCCGTCAAACAGAAAGTGCACCGAGCCCGAAGGCAAGGTTGAGTTCTGAACGACGCGACTGACTTCAATCGTTTCCCTTTCAGCAATTTCACGTACTGTTTAACTCTCTTTCCAAAGTGCTTTTCATCTTTCCCTCACGGTACTTGTTCGCTATCGGTCTCTCGCCAATATTTAGCTTTAGATGGAATTTACCACCCATTTTGAGCTGCATTCCCAAACAACTCGACTCTTTGAGAGCGCATCACAAAGCATCGGTAGTCCGTGTCAAAGACGGGATTCTCACCCTCTATGACGCTCTGTTCCAAGAGACTTGTACAC	ITS: 100% with CBS 280.73 LSU: 100% with CBS 280.73
*Trametes betulina* (syn. *Lenzites betulinus*) Specimen #28		
GCTGGCCTTCCGAGGCATGTGCACGCCCTGCTCATCCCACTCTACCCCTGTGCACTTACTGTAGGTCGGCGTGGGTTTCTAGCCTCCGGGTTTGAAGCATTCTGCTGGCCTATGTACATTTATAAACACTTTAAAGTAACAGAATGTAAACGCGTCTAACGCATTTTAATACAACTTTCAGCAACGGATCTCTTGGCTCTCGCATCGATGAAGAACGCAGCGAAATGCGATAAGTAATGTGAATTGCAGAATTCAGTGAATCATCGAATCTTTGAACGCACCTTGCGCTCCTTGGTATTCCGAGGAGCATGCCTGTTTGAGTGTCATGGAATTCTCAACCCATAAATCCTTGTGATGTATGGGCTTGGATTTGGAGGCTTGCTGGTCCCTTTCGGGATCGGCTCCTCTTGAATATATTAGCTTGATTCCGTGCGGATCGGCTCTCAGTGTGATAATTATCTACGCTGTGACCGTGAAGCGTTTGGCGAGCTTCTAACCGTCCTTTTTGGACAAACCTTATGACATCTGACCTCAAATCAGGTAGGACTACCCGC	GTAACTGCGAGTGAAGCGGGAAAAGCTCAAATTTAAAATCTGGCGGTCTTTGGCCGTCCGAGTTGTAGTCTGGAGAAGTGCTTTCCGCGCTGGACCGTGTACAAGTCTCTTGGAACAGAGCGTCATAGAGGGTGAGAATCCCGTCTTTGACACGGACTACCAGTGCTTTGTGATGCGCTCTCAAAGAGTCGAGTTGTTTGGGAATGCAGCTCAAAATGGGTGGTGAATTCCATCTAAAGCTAAATATTGGCGAGAGACCGATAGCGAACAAGTACCGTGAGGGAAAGATGAAAAGCACTTTGGAAAGAGAGTTAAACAGTACGTGAAATTGCTGAAAGGGAAACGCTTGAAGTCAGTCGCGTTGTCCAGAACTCAGCCTTGCTTCGGCTTGGTGCATTTTCTGGGCGACGGGCCAGCATCGATTTTGACCGTCGGAAAAGGGTTGAAGGAATGTGGCACCTTCGGGTGTGTTATAGCCTTCAGCCGCATACGACGGTTGGGATCGAGGAACGCAGCGCGCCTTATGGCTGGGGTTCGCCCACATTCGCGCTTAGGATGCTGGCATAATGGCTTTAAACGACCCGTCTTGAAACACGGACCAAGGAGTCTAACATGCCTGCGAGTGTTTGGGTGGAAAACCCGAGCGCGTAATGAAAGTGAAAGTTGAGACCTCTGTCGTGGAGGGCATCGACGCCCGGACCTGACGTTCTCTGACGGATCCGCGGTAGAGCATGTATGTTGGGACCCGAAAGATGGTGAACTATGCCTGAATAGGGTGAAGCCAGAGGAAACTCTGGTGGAGGCTCGTAGCGATTCTGACGTGCAAATCGATCGTCAAATTTGGGTATAGGGGCGAAAGACTAATCGAACCATCTAGTAGCTGGTTCCTGCCGAAGTTTCCCTCAGGATAGCAGAAACTCATATCA	ITS: 100% with OM530254 LSU: 100% with OL685336
*Trametes gibbosa* Specimen #22		
CTTCCGAGGCATGTGCACGCCCTGCTCATCCACTCTACACCTGTGCACTTACTGTAGGTCTGCGTGGGTTTCTAGCCTCCGGGTTGGGAGCATTCTGCAGGCTTATGTATACTATAAACACTTTAAAGTAACAGAATGTAAACGCGTCTAACGCATTTTAATACAACTTTCAGCAACGGATCTCTTGGCTCTCGCATCGATGAAGAACGCAGCGAAATGCGATAAGTAATGTGAATTGCAGAATTCAGTGAATCATCGAATCTTTGAACGCACCTTGCGCTCCTTGGTATTCCGAGGAGCATGCCTGTTTGAGTGTCATGAAATTCTCAACCCATAAATCCTTGTGGAAGTATGGGCTTGGATTTGGAGGTTTGCTGGGTCCCCTGGGGTCCGGCTCCTCTCGAACGCATTAGCTTGATTCCGTGCGGATCGGCTCTCAGTGTGATAATTATCTACGCTGTGACCGTGAAGCGTTTGGCGAGCTTCCAACCGTCTTTTTTGGACAACCTTATGACATCTGACCTCAAATCAGGTAGGACTACCCGCTGAACTTAAGCATATCATAAACCCGGAAAAAAAAGAAAATTAAAAAAGTTTTGAAATGG	TAGGTTAAGGTTGTTTCAACCCCAAGGCCTCTAATCATTCGCTTTACCACATAAATCTGATATGAGTTTCTGCTATCCTGAGGGAAACTTCGGCAGGAACCAGCTACTAGATGGTTCGATTAGTCTTTCGCCCCTATACCCAAATTTGACGATCGATTTGCACGTCAGAATCGCTACGAGCCTCCACCAGAGTTTCCTCTGGCTTCACCCTATTCAGGCATAGTTCACCATCTTTCGGGTCCCAACATACATGCTCTACCGCGGATCCGTCAGAGAACGTCAGGTCCGGGCGTCGATGCCCTCCACGACAGAGGTCTCAACTTTCACTTTCATTACGCGCTCGGGTTTTCCACCCAAACACTCGCAGGCATGTTAGACTCCTTGGTCCGTGTTTCAAGACGGGTCGTTTAAAGCCATTATGCCAGCATCCTAAGCACGAATGTGGGCGAACCCCAGCCATAAGGCGTGCTGCGTTCCTCGATCCCAACCGCCGTATGCGACTGAAGGCTATAACACACCCGAAGGTGCCACATTCCCTCAGCCCTTTTCCGACGGTCAAAATCGATGCTGGCCCGTCGCCCGGAAAATGCACCAAGCCGAAGCAAGGCTGAGTTCCGGACAACGCGACTGACTTCAAGCGTTTCCCTTTCAGCAATTTCACGTACTGTTTAACTCTCTTTCCAAAGTGCTTTTCATCTTTCCCTCACGGTACTTGTTCGCTATCGGTCTCTCGCCAATATTTAGCTTTAGATGGAATTCACCACCCATTTTGAGCTGCATTCCCAAACAACTCGACTCTTTGAGAGCGCATCACAAAGCACTGGTAGTCCATGTCAAAGACGGGATTCTCACCCTCTATGACGCTCTGTTCCAAGAGACTTGTACACGGTCCAGCGCGGA	ITS: 100% with EU162057 LSU: 99.88% with KC176329 and 100% with MF115828 (GenBank)
*Trametes versicolor* Specimen #30		
GCTGGCCTTCCGAGGCATGTGCACGCTCTGCTCATCCACTCTACCCCTGTGCACTTACTGTAGGTTGGCGTGGGCTCCTTAACGGGAGCATTCTGCCGGCCTATGTATACTACAAACACTTTAAAGTATCAGAATGTAAACGCGTCTAACGCATCTATAATACAACTTTTAGCAACGGATCTCTTGGCTCTCGCATCGATGAAGAACGCAGCGAAATGCGATAAGTAATGTGAATTGCAGAATTCAGTGAATCATCGAATCTTTGAACGCACCTTGCGCTCCTTGGTATTCCGAGGAGCATGCCTGTTTGAGTGTCATGGAATTCTCAACTTATAAATCCTTGTGATCTATAAGCTTGGACTTGGAGGCTTGCTGGCCCTTGTTGGTCGGCTCCTCTTGAATGCATTAGCTCGATTCCGTACGGATCGGCTCTCAGTGTGATAATTGTCTACGCTGTGACCGTGAAGTGTTTTGGCGAGCTTCTAACCGTCCATTAGGACAATTTTTTAACATCTGACCTCAAATCAGGTAGGACTACCCGCTGAACTTAAGCATATCATAAAACGAGGGAAAAAAAAAAAATTAAACAAGTTTTGAAACGAGTTGGAAGCTGGCCTTCCAAGGCAGGTGCAGCTCTGCTAAACCACTCTACCCCAGTGCACTTACCGTAGGTAGGCGTGGGCCCCTAAACGGGAGCCATTCTCCGGCCTATGTATACAACAAACACTTTAAAGTATCAAAATGTAAACCGTCTAAGCACCTTTAAAACAACTTTTAGCACCGAAACTTTGGGCTCTGCATCGATAAAAAAAGCACCGAAATGCGATAAGTAAGGTGAAATTGCAAAAATTCTTGAAACCCCCAATCTTTAAACCCCTGGGGTTCCGGGGTATTCCCAGG	CAAGCGACGGCTCGTTCTTACATATTTAAAGTTTGAGAATAGGTTAAGGTTGTTTCAACCCCAAGGCCTCTAATCATTCGCTTTACCACATAAATCTGATATGAGTTTCTGCTATCCTGAGGGAAACTTCGGCAGGAACCAGCTACTAGATGGTTCGATTAGTCTTTCGCCCCTATACCCAAATTTGACGATCGATTTGCACGTCAGAATCGCTACGAGCCTCCACCAGAGTTTCCTCTGGCTTCACCCTATTCAGGCATAGTTCACCATCTTTCGGGTCCCAACATACATGCTCTACCGCGGATCCTTCAGAAAACGTCAGGTCCGGGCGTCGATGCCCTCCACGACAGAGGTCTCAACTTTCACTTTCATTACGCGCTCGGGTTTTCCACCCAAACACTCGCAGGCATGTTAGACTCCTTGGTCCGTGTTTCAAGACGGGTCGTTTAAAGCCATTATGCCAGCATCCTAAGCGCGAATGTGGGCGAACCCCAGCCATAAGGCGCGCTGCGTTCCTCGATCCCAACCGCTGTATGCGACTGAAGGCTATAACACACCCGAAGGTGCCACATTCCTCCAGCCCTTTTCCAGCGGTCAAAATCGATGCTGGCCCGTCAACCGGAAAGTGCACTAAGCCGAAGCAAAGCTGAGTTCCGGACGACGCGACTGACTTCAAGCGTTTCCCTTTTAGCAATTTCACGTACTGTTTAACTCTCTTTCCAAAGTGCTTTTCATCTTTCCCTCACGGTACTTGTTCGCTATCGGTCTCTCGCCAATATTTAGCTTTAGAAGGAATTCACCTCCCATTTTGCGCTGCATTCCCAAACAACGCGACTCTTTGAGAGCGCATCACAAAGCATTGGTAGTCCGTGTCAAAGACGGGATTCTCACCCTCTATGACGCTCTGTTCCAAGAGACTTGTACACGGTCCAACGCGGAAGACGCTTCTCCAGACTACAACTCGGACGGC	ITS: 98.62% with KC176302 LSU: 100% with CBS 294.33 and 100% with ON402850 (GenBank)
TEF1 sequence		Similarity
TCTCGACGGACTTAACCTCAGTGGTCACGTTCGAGGGCGCGAAGGTGACGACCATGCCGGCCTTGATGACACCGGTCTCGACACGGCCGACGGGCACCGTGCCAATACCGCCGATCTTGTAGACATCCTGGAGGGGGAGACGGAGGGGCTTGTCGGAGGGACGGACGGGGGGCTCGATGGCGTCGATGGCGTCGAGGAGCGTCTTGCCCTTGACAACACCAGCCTTGGTCTCCTTGGTCCAGCCCTTGTACCAGGTCATGCTGAGAGAGCGTCAGCACGATGTGCCTGAAGAATCCGAGAGCATATACGCACTTGGCAGACTCCTCCAACATGTTGTCGCCGTGCCATCCGGAGATGGGGACGAACGCGACCGCCTTCGGGTTGTAGCCGACCTTCTTGATGAAGGTCGACGTCTCCTTGATGATCTCGTTGAAACGGTCCTCGGACCACTATATGCGCGTAGTTAGTATAGCACGCAATGCTGATAGCGCGCGATAACGAACCTTCGTGGTGTCCATCTTGTTGACGGCGACGATGAGCTGCCTGACACCGAGGGTGAAGGCAAGGAGAGCGTGCTCGCGGGTCTGGCCATCCTTGGAGATACCAGCCTCGAACTCACCGGTACCAGCGGCGATGATGAGGATAGCGCAGTCAGCCTGCGAGGTACCGGTGATCATGTT		TEF1: 96.91% with JN164878 (GenBank)

The TEF1 region sequence was also used to identify specimen #30

**Table 2:** Biomass, viscosity, consistency index ($K$), flow behaviour index ($n$) and pH of mono- and co-cultures after 7 days of growth in MEB (mean ± 95% confidence interval)

Species	Biomass (g L^−1^)	Viscosity (mPa·s)	$K$(mPa·s^*n*^)	$n$	pH
Mono-cultures					
*Bjerkandera adusta*	3.94 ± 0.62^a^	1.13 ± 0.03^a^	n/a	1.00 ± 0.02^ab^	5.4 ± 0.24^ab^
*Chondrostereum purpureum*	4.87 ± 0.37^ab^	1.11 ± 0.02^ab^	n/a	0.98 ± 0.01^a^	6.8 ± 0.07^c^
*Fomes inzengae*	2.43 ± 0.22^c^	1.13 ± 0.08^a^	n/a	0.97 ± 0.03^ab^	5.3 ± 0.06^a^
*Ganoderma australe*	4.78 ± 0.81^abde^	n/a	40.8 ± 26.1^ab^	0.57 ± 0.10^c^	5.0 ± 0.27^ad^
*Ganoderma resinaceum*	7.58 ± 0.18^ fg^	1.09 ± 0.04^ab^	n/a	0.99 ± 0.01^ab^	5.6 ± 0.12^b^
*Phlebia tremellosa*	5.25 ± 0.30^abd^	n/a	30.1 ± 12.5^ab^	0.58 ± 0.09^c^	4.8 ± 0.13^d^
*Pleurotus ostreatus*	5.31 ± 0.28^bd^	1.02 ± 0.01^b^	n/a	1.00 ± 0.00^b^	7.1 ± 0.32^c^
*Schizophyllum commune*	8.62 ± 0.23^ h^	n/a	491.4 ± 134.2^c^	0.24 ± 0.04^d^	6.2 ± 0.10^e^
*Trametes betulina*	6.94 ± 0.30^i^	1.03 ± 0.05^b^	n/a	1.02 ± 0.02^ab^	3.5 ± 0.06^f^
*Trametes gibbosa*	5.95 ± 0.44^de^	n/a	5.8 ± 0.8^b^	0.79 ± 0.02^f^	4.7 ± 0.12^d^
*Trametes versicolor*	5.93 ± 0.14^e^	n/a	48.2 ± 4.7^ab^	0.52 ± 0.02^c^	4.6 ± 0.02^d^
Co-cultures					
*G. resinaceum* and *T. betulina*	4.74 ± 0.15^ab^	1.08 ± 0.02^ab^	n/a	1.00 ± 0.02^ab^	4.4 ± 0.17^d^
*S. commune* and *G. resinaceum*	8.03 ± 0.19^f^	n/a	121.4 ± 22.6^a^	0.36 ± 0.02^e^	5.9 ± 0.14^b^
*T. betulina* and *S. commune*	7.39 ± 0.17^gi^	n/a	29.9 ± 3.2^ab^	0.53 ± 0.01^c^	5.6 ± 0.04^b^

MEB medium has a viscosity of 1.27 ± 0.08 mPa·s ($n$ = 0.99 ± 0.01) and a pH of 5.1 ± 0.01. Letters in superscript indicate statistically significant differences within a column

The rheological properties of spent medium from the 7-day-old fungal cultures showed either Newtonian (*Bjerkandera adusta*, *Chondrostereum purpureum*, *F. inzengae*, *G.* *resinaceum*, *P. ostreatus*, *T. betulina*) or non-Newtonian shear-thinning (*Ganoderma australe, Phlebia tremellosa*, *S. commune*, *Trametes gibbosa*, *Trametes versicolor*) behaviour (Fig. S1). While the Newtonian behaviour suggests uniform viscosity regardless of shear rate, the non-Newtonian shear-thinning behaviour indicates a decrease in viscosity with increasing shear rates (Mezger 2014). For example, *G. resinaceum* and *T.* *betulina* displayed Newtonian behaviour with viscosities of 1.09 and 1.03 mPa·s, respectively, both lower than the viscosity of MEB itself (1.27 mPa·s) but higher than dH_2_O (0.89 mPa·s) (Table 2). For species exhibiting shear-thinning behaviour, *S. commune* had the highest consistency index ($K$) of 491.4 mPa·s^*n*^ and most pronounced shear-thinning behaviour as indicated by the lowest flow behaviour index ($n$). Co-cultures of *S. commune* with either *G. resinaceum* or *T.* *betulina* were less viscous at low shear rates 121.4 and 29.9 mPa·s^*n*^, respectively.

### Material properties of PMMs

**Fig. 1:**
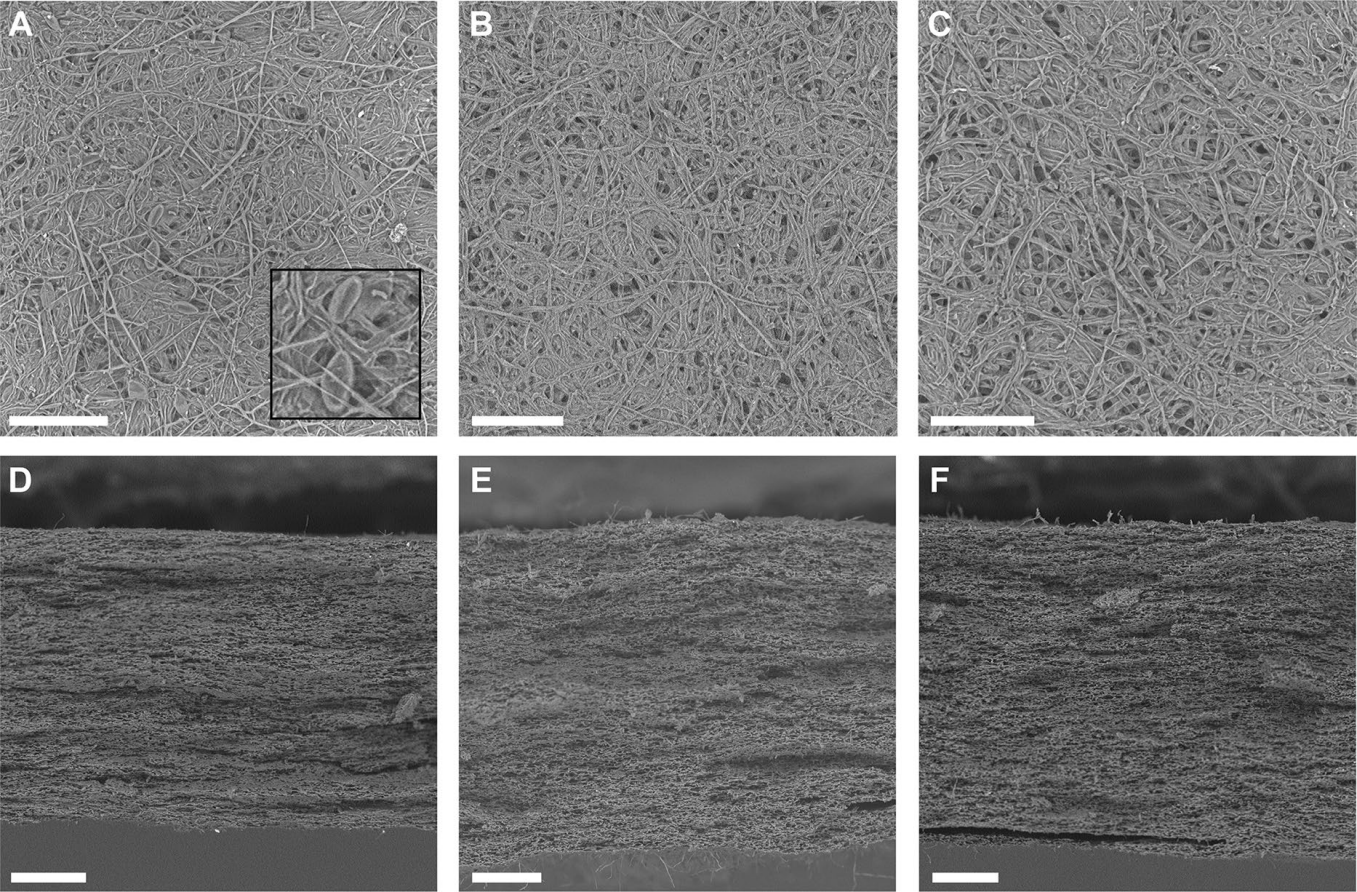
Scanning electron microscopy of the surface (**A**–**C**; scale bar 50 μm) and longitudinal cross section (**D**–**F**; scale bar 100 μm) of *G. resinaceum* (**A**, **D**), *S. commune* (**B**, **E**) and *T. betulina* (**C**, **F**) PMMs. The inset in **A** shows an enlarged view of chlamydospores present in *G.* *resinaceum* PMM

PMMs were produced from mono-cultures, mixed-cultures, and co-cultures of *G.* *resinaceum*, *S. commune* and *T.* *betulina* because these fungi produced most biomass as mono-cultures (see above). The hyphal diameter of *G.* *resinaceum*, *S. commune* and *T.* *betulina* was 2.2, 2.5 and 3.1 μm, respectively. All three species grew as pellets, albeit of varying size and shape. Pellets of *G.* *resinaceum* and *T.* *betulina* were spherical, whereas pellets of *S. commune* were characterised as non-spherical (hairy) (Fig. S2). Forming films by vacuum filtration directly from pellets was possible with *S. commune*, however not with *G.* *resinaceum* and *T.* *betulina*, resulting in non-uniform PMM sheets after drying. For this reason, pellet biomass was blended before drying. Sheets of blended biomass of *G. resinaceum*, *S. commune* and *T. betulina* shrank by 34.4, 27.4, and 33.9%, respectively, during drying (Table 3). SEM revealed the presence of chlamydospores in the *G. resinaceum* mono-culture PMM (Fig. 1A) and its mixed PMMs, but not in the sheets of the *G. resinaceum* co-cultures.

**Table 3:** Material properties of mono-cultures of *G. resinaceum* (GR), *S. commune* (SC), and *T. betulina* (TB) and their mixed- and co-cultures

Species	Mass (g)	Shrinkage (%)	Thickness (mm)	Density (kg m^−3^)	σ (MPa)	ε (%)	*E* (MPa)	*n*
Mono-cultures								
GR	0.23 ± 0.01^ab^	34.4 ± 2.7^a^	0.49 ± 0.02^a^	730 ± 14^a^	5.2 ± 0.7^a^	0.9 ± 0.1^a^	562 ± 98^ab^	44
SC	0.22 ± 0.01^ac^	27.4 ± 2.5^bc^	0.61 ± 0.03^bc^	550 ± 30^ cd^	6.3 ± 0.7^a^	1.3 ± 0.1^c^	546 ± 96^ab^	36
TB	0.24 ± 0.01^bd^	33.9 ± 4.3^abc^	0.68 ± 0.03^d^	545 ± 23^c^	5.9 ± 0.9^a^	0.9 ± 0.1^a^	677 ± 191^ab^	29
Mixed-cultures								
GR + TB	0.22 ± 0.00^ac^	34.0 ± 2.7^a^	0.55 ± 0.01^b^	628 ± 9^b^	4.9 ± 0.5^a^	1.0 ± 0.1^ab^	430 ± 51^a^	30
SC + GR	0.22 ± 0.02^abc^	31.6 ± 9.6^abc^	0.57 ± 0.08^abcd^	604 ± 45^bc^	4.5 ± 1.2^a^	0.9 ± 0.1^a^	408 ± 126^ab^	16
TB + SC	0.20 ± 0.01^c^	23.9 ± 12.1^abc^	0.53 ± 0.05^ab^	583 ± 32^bc^	4.6 ± 0.9^a^	0.8 ± 0.1^a^	503 ± 105^ab^	24
Co-cultures								
GR and TB	0.25 ± 0.01^d^	36.7 ± 4.8^ab^	0.63 ± 0.06^bcd^	621 ± 43^bc^	6.2 ± 1.0^a^	0.8 ± 0.1^a^	710 ± 152^b^	16
SC and GR	0.22 ± 0.01^ac^	26.2 ± 3.4^c^	0.67 ± 0.03^ cd^	492 ± 16^de^	6.0 ± 0.5^a^	1.6 ± 0.2^c^	475 ± 90^ab^	23
TB and SC	0.23 ± 0.01^ab^	31.7 ± 4.8^abc^	0.75 ± 0.03^e^	467 ± 28^e^	6.2 ± 0.9^a^	1.2 ± 0.1^bc^	527 ± 169^ab^	20

σ, ε, and *E* indicate maximum tensile strength, elongation at break, and Young’s modulus, respectively (mean ± 95% confidence interval). The number of replicates is shown (*n*) except for shrinkage (*n* = 6 − 19). Letters in superscript indicate statistically significant differences within a column

The mono-culture *G. resinaceum* PMM had a significantly higher density (730 kg m^−3^) compared to the mono-culture PMMs of *S. commune* and *T. betulina* (550 and 545 kg m^−3^, respectively). Based on visual inspection of the SEM images (Fig. 1), the amount of air voids in the PMMs appeared similar. Density of the mixed PMMs as well as the co-culture of *G. resinaceum* and *T. betulina* was similar to the mono-culture PMMs of *S. commune* and *T. betulina* (ranging between 583 and 628 kg m^−3^), while the co-cultures of *S. commune* with *G. resinaceum* or *T.* *betulina* was most similar to the *S. commune* mono-cultures (467–492 kg m^−3^).

The maximum tensile strength of the materials produced from the different species and their combinations, whether derived from mono-culture, mixed-, or co-culture conditions, did not show significant differences and ranged from 4.5 to 6.3 MPa (Table 3). Yet, the maximum tensile strength of the PMMs of the three mixed-cultures was significantly lower compared to the PMMs of the three mono-cultures and the three co-cultures. The elongation at break of the different PMMs was generally low and ranged between 0.8 and 1.6%. The *S. commune* mono-culture and the co-culture of *S. commune* and *G. resinaceum* showed the highest elongation at break of 1.3 and 1.6%, respectively. The Youngs modulus also showed relatively small differences ranging between 408 and 710 MPa. Only the Youngs modulus of the co-culture of *S. commune* and *G. resinaceum* (430 MPa) was significantly different from that of the co-culture of *G. resinaceum* and *T. betulina* (710 MPa).

### Water absorption and contact angle of PMMs

**Fig. 2:**
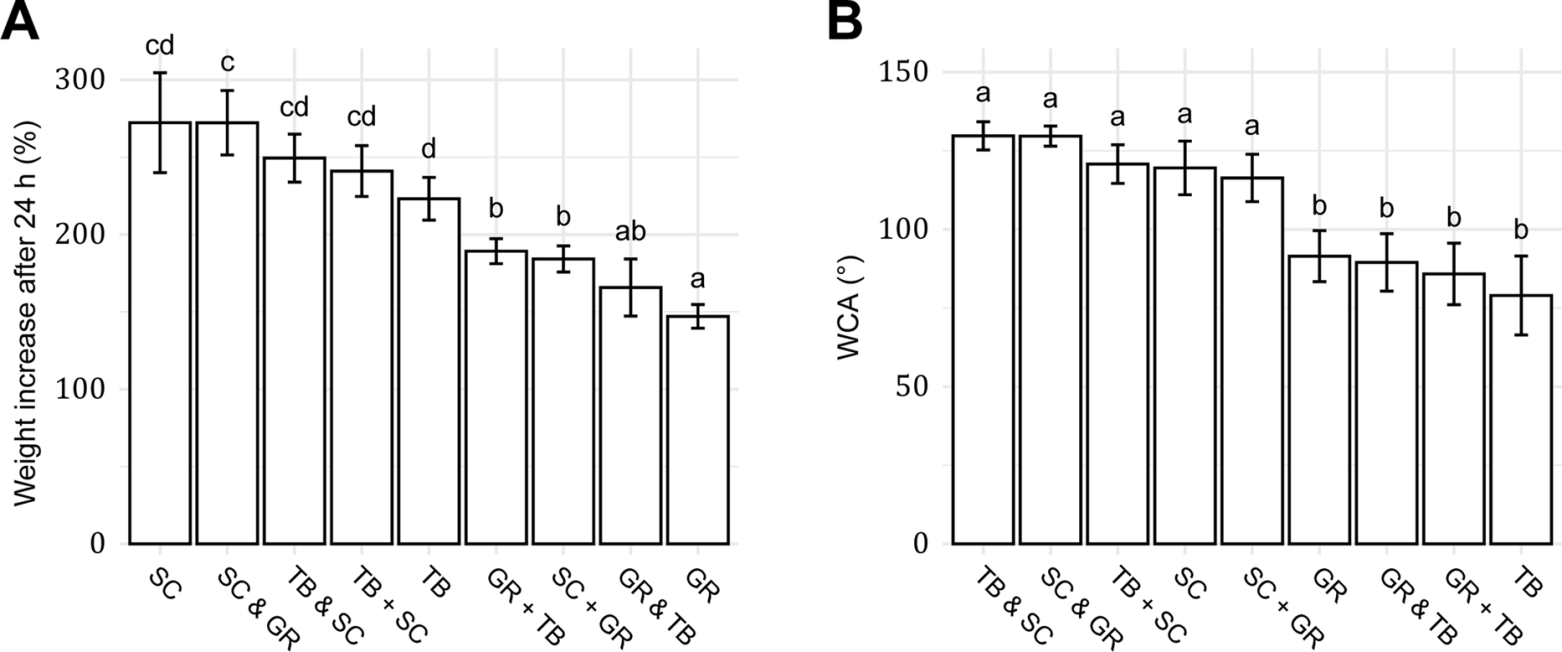
Weight increase (wt %) after 24 h submersion in dH_2_O (*n* = 12 − 29) **A** and water contact angle measurements (*n* = 12 − 39) **B** for mono-, mixed- (indicated with +), and co-cultures (indicated with and) of PMMs from *G. resinaceum* (GR), *S. commune* (SC), and *T. betulina* (TB). Data are presented as mean ± 95% confidence interval and letters indicate statistically significant differences between mono-, mixed- and co-cultures

The PMM of *G. resinaceum* absorbed significantly less water during 24 h, increasing in weight by 147%, compared to the 272 and 223% weight increase observed in *S. commune* and *T.* *betulina*, respectively (Fig. 2A). PMMs of the latter two fungi mixed with biomass of *G. resinaceum* had a reduced water absorption compared to their respective mono-culture PMMs with values of 189 and 184%, respectively. In addition, the co-culture of *G.* *resinaceum* and *T.* *betulina* (166%) had a similar water uptake as the *G. resinaceum* mono-culture PMM after 24 h submersion. Together, *G. resinaceum* PMMs showed the lowest water uptake (Fig. S3). On the other hand, the *S. commune* mono-, co- and mixed-PMMs showed the highest WCA ranging between 116 ± 8 and 130 ± 4° (Fig. 2B), while PMMs without *S. commune* biomass showed a WCA ranging between 79 and 91°.

## Discussion

In this study, 10 mushroom-forming fungi were isolated, identified and grown in liquid shaken MEB cultures at 30 °C and 200 rpm. The top three biomass producing species (*G. resinaceum*, *S. commune* and *T. betulina*) were selected for further processing into PMMs using mono- and co-cultures, as well as by mixing the mycelium from mono-cultures (so called mixed-cultures). The use of *G.* *resinaceum* and *T.* *betulina* as PMMs has been described by others, but no mechanical properties have been reported thus far (Adamatzky et al. 2021; Cartabia et al. 2021; Elsacker et al. 2023). By contrast, mechanical properties of *S. commune* PMMs have been described in the literature (Appels et al. 2018, 2020; d’Errico et al. 2024; van den Brandhof et al. 2024). Importantly, methods to produce PMMs differ, with liquid-state surface fermentation and solid-state fermentation being static, while here we describe liquid-state fermentation under agitated conditions.

Identification of the specimens was successful for 9 out of 10 specimens using the ITS and LSU regions. However, specimen #30 was more difficult to identify because *Trametes versicolor* is part of a species complex. Therefore, the TEF1 region was sequenced as well (Carlson et al. 2014), confirming that this strain belonged to *T. versicolor.* Screening isolates in liquid shaken cultures revealed varying biomass production of the different isolates, with *S. commune* yielding the highest biomass at 8.62 g L^−1^, while *F.* *inzengae* produced the lowest at 2.43 g L^−1^. Co-culture combinations of the top three species generally exhibited intermediate biomass yields compared to their respective mono-cultures. Additionally, rheological characterization of spent medium showed either Newtonian or shear-thinning behaviour. *G.* *resinaceum* and *T.* *betulina* displayed Newtonian behaviour with lower viscosities than the growth medium, whereas *S. commune* had the strongest shear-thinning effect, which can be attributed to its production of the extracellular polysaccharide schizophyllan (Steiner et al. 1988). Low viscosity is favorable for liquid-state fermentation under agitated conditions, as it makes separation of biomass from the culture easier. In combination with relatively high biomass production, this makes *G.* *resinaceum* and *T.* *betulina* good candidates for PMMs using liquid-state fermentation.

Overall, PMMs produced using liquid shaking cultures from mono- and co-cultures, as well as by mixing the mycelium from mono-cultures, did not result in a significant difference in maximum tensile strength. However, *S. commune* achieved the highest tensile strength (6.3 MPa) despite having a lower density (550 kg m^−3^) than *G. resinaceum*, which exhibited the highest measured density (730 kg m^−3^) but a lower tensile strength (5.2 MPa). With a density in a similar range (749 kg m^−3^), *S. commune* reached a higher tensile strength of 10.3 MPa after two wetting and drying cycles (van den Brandhof et al. 2024). Blending the pellet biomass was essential to form films with *G.* *resinaceum* and *T.* *betulina*, and appears to enhance the mechanical properties of *S. commune*, as seen in our study, where it resulted in a tensile strength of 6.3 MPa with a density of 550 kg m^−3^. By contrast, untreated films produced from unblended *S. commune* exhibited lower tensile strength of 5.0 MPa (Appels et al. 2020) and 3.0 MPa (van den Brandhof et al. 2024), with densities of 587 kg m^−3^ and 451 kg m^−3^, respectively.

SEM did not reveal why the *G. resinaceum* mono-culture PMM had a higher density than the *S. commune* and the *T.* *betulina* mono-culture PMMs, with no obvious difference in air voids observed between the three PMMs. However, it is important to note that the density of *G. resinaceum* PMM was only 1.3-fold higher than that of both *T. betulina* and *S. commune*, whereas in a previous study, SEM visually confirmed a more pronounced higher density in the PMM derived from *S. commune* Δsc3, which was threefold higher compared to the wild-type (Appels et al. 2018)*.* Similarly, SEM after three wetting and drying cycles showed a clear higher density, with a twofold increase compared to PMM before the wetting and drying cycles (van den Brandhof et al. 2024). SEM did show the presence of chlamydospores in the *G. resinaceum* mono- and mixed-culture PMMs, similar to previous observations in *G. lucidum* (Elsacker et al. 2023). These chlamydospores are more resistant to high temperature stress and therefore heat inactivation of the PMM of *G. resinaceum* resulting from liquid shaken cultures should be confirmed by plating.

To the best of our knowledge, this is the first reported use of co-culturing two fungal species to produce PMMs. Co-culturing of fungi is mainly studied to improve biosynthesis of secondary metabolites (Knowles et al. 2022). Little to no mechanical advantage was observed in the co-culture material compared to the mono-culture material, as no significant difference was found in maximum tensile strength, while elongation at break showed a significant but minor absolute difference. Apparently, co-culturing of *G. resinaceum*, *S. commune* and *T. betulina* did not induce stress responses that resulted for instance in a changed mycelium network or a changed cell wall composition; both of which are expected to impact the mechanical properties of the PMMs.

It should be noted that we used a single cultivation condition (i.e. growth in MEB at 30 °C and 200 rpm) for all 11 fungi. Most likely, this is not an optimal growth condition for the majority of these fungi. Therefore, future research should assess the properties of the mycelium of mono-, mixed- and co-cultures under optimal growth conditions for each of the fungi. This should reveal whether also under these conditions results indicate that the species of mushroom forming fungi does not have a major impact on PMM properties of biomass from liquid shaken cultures.

## Supplementary Information

Below is the link to the electronic supplementary material.

Supplementary file1 (DOCX 10145 kb)

## Data Availability

Data will be made available on request.
