# Supplementary material for: Characterization of pure mycelium materials from different mushroom-forming fungi
Source: Antonie Van Leeuwenhoek. 2025 Jul 23;118(9):121. doi: 10.1007/s10482-025-02133-5 (PMC12287172; doi:10.1007/s10482-025-02133-5)
Supplement: Supplementary file 1 — Supplementary file1 (DOCX 10145 kb) [file 10482_2025_2133_MOESM1_ESM.docx]

**Supplementary Information**

**Characterization of pure mycelium materials from different mushroom-forming fungi**

Jeroen G. van den Brandhof, Noortje Hansen, Chen Hou, Sander C. Broers, Martin Tegelaar & Han A. B. Wösten*

**Table S1.** Primers used in this study.

| Primer | Sequence |
| --- | --- |
| ITS1 | TCCGTAGGTGAACCTGCGG |
| ITS4 | TCCTCCGCTTATTGATATGC |
| LR0R | ACCCGCTGAACTTAAGC |
| LR6 | CGCCAGTTCTGCTTACC |
| EF1-1018F | GAYTTCATCAAGAACATGAT |
| EF1-1620R | GACGTTGAADCCRACRTTGTC |

**
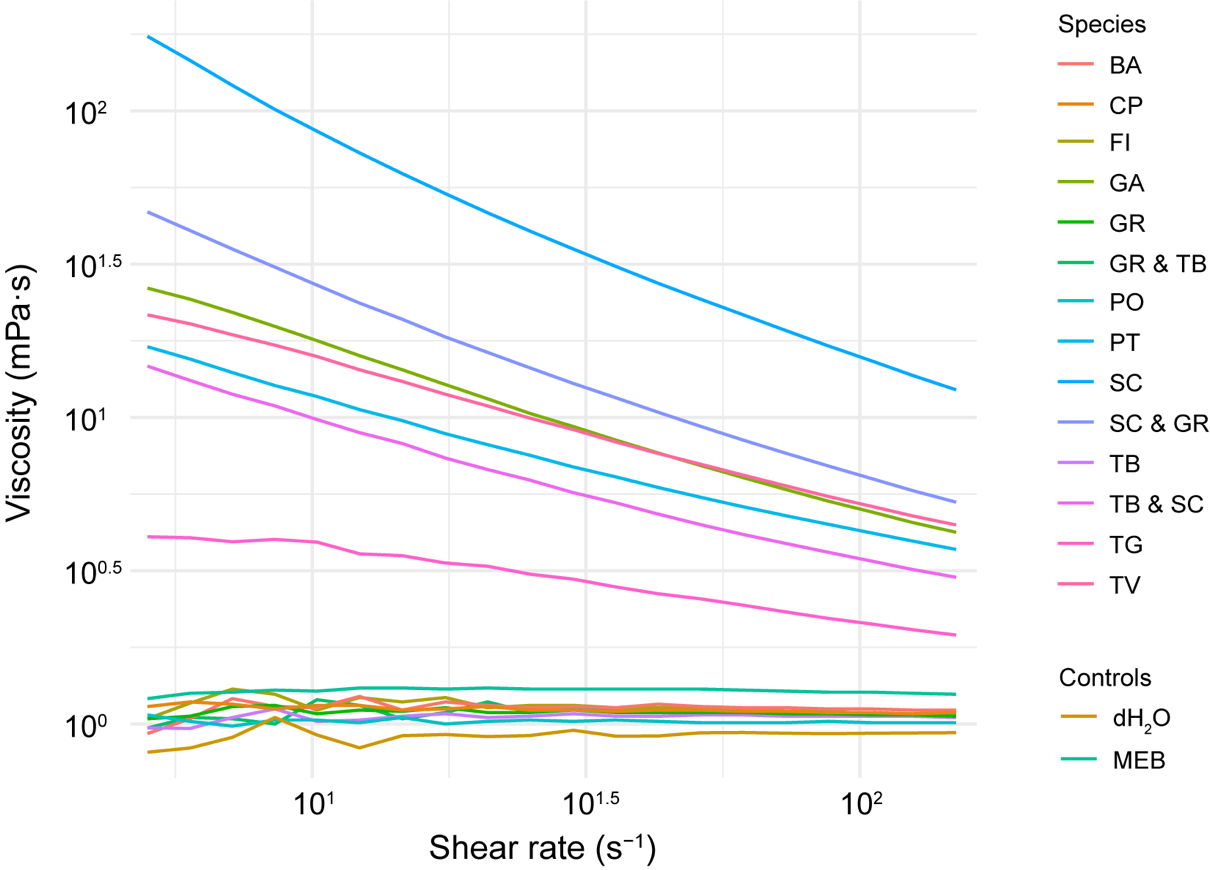
**

**Fig. S1.** Representative rheology curves of spent medium of 11 species grown as mono-culture and 3 co-cultures (indicated with &) after 7 days of growth in 250 ml Erlenmeyers with 100 ml MEB. dH_2_O and MEB served as controls.

**
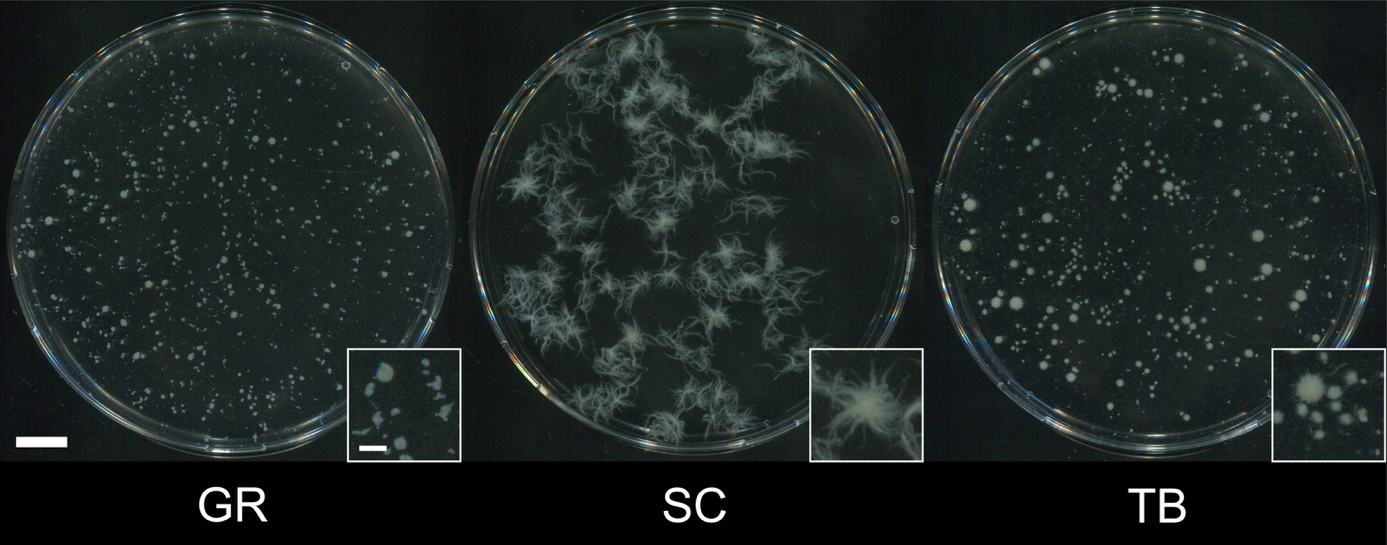
**

**Fig. S2.** Pellet morphology of *G. resinaceum* (GR), *S. commune* (SC) and *T. betulina* (TB) in 85 mm diameter Petri dishes after 7 days of growth in 2 L Erlenmeyers with 1200 ml MEB. Bars represent 1 cm and 2 mm in the case of the Petri dishes and the insets, respectively.

**
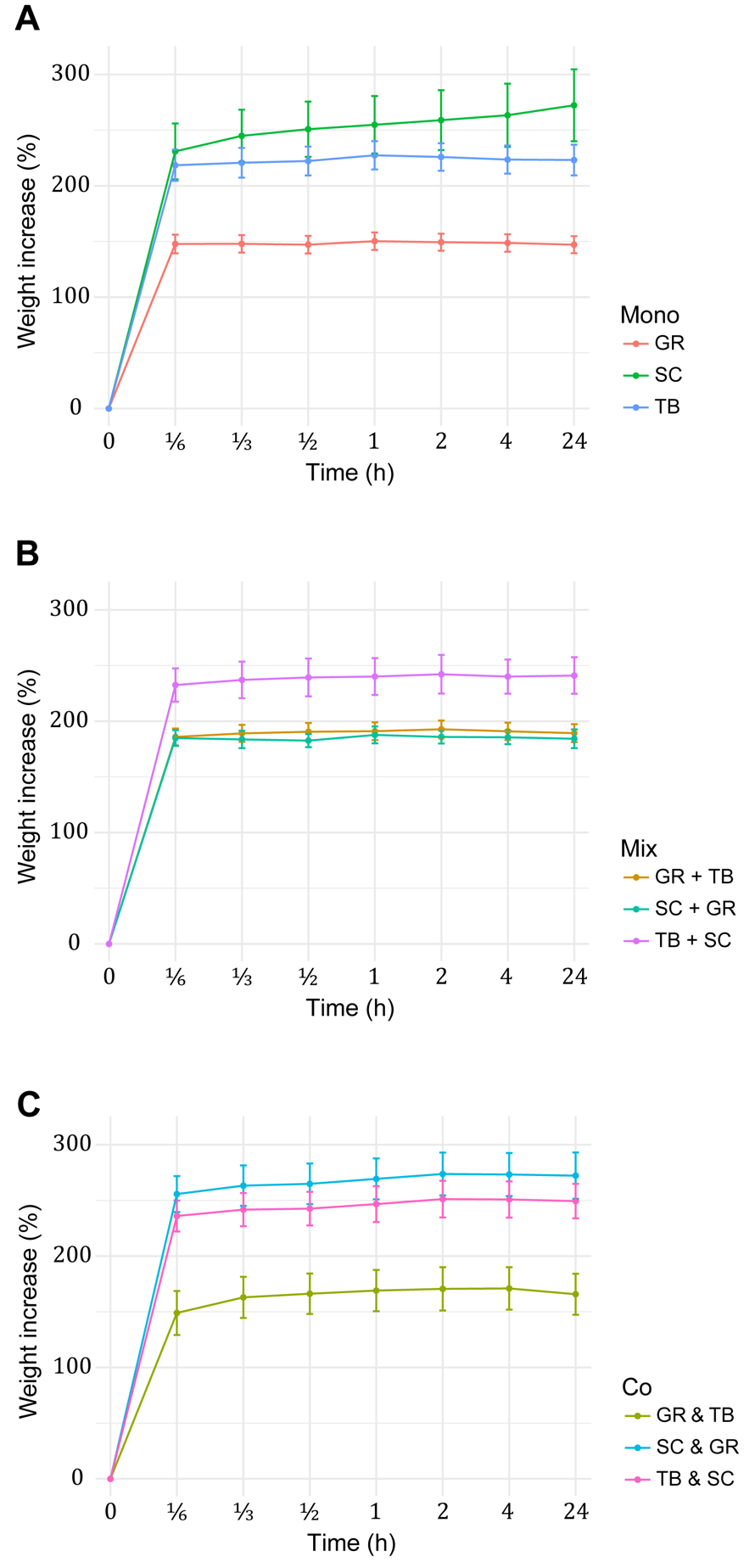
**

**Fig. S3.** Weight increase (wt %) over time after submersion in dH_2_O (*n* = 12−29) for mono- (**A**), mixed- (**B**), and co-cultures (**C**) of PMMs from G. resinaceum (GR), S. commune (SC), and T. betulina (TB). Data are presented as mean ± 95 % confidence interval.
